# Effectiviness of a crisis comunity-based program in primary mental health care

**DOI:** 10.1192/j.eurpsy.2023.1001

**Published:** 2023-07-19

**Authors:** S. Lakis, S. Mansilla, B. Patrizzi, C. Teixidó, J. Vegué, A. Plaza

**Affiliations:** PSYCHIATRY, CPB SERVEIS SALUT MENTAL, BARCELONA, Spain

## Abstract

**Introduction:**

A crisis is defined as a disruption in equilibrium at the failure of own personal resources which results in important distress and functional impairment. Particularly after Covid-19 pandemic we have been attending an increased number of this kind of consultations in the mental health services.

**Objectives:**

To expose a new mental health program called PAIC (Ambulatory Intensive Community Program) started in March 2019 and addressed to people who are suffering a crises period, and to evaluate its effectiveness. The aim of this program is to reach prior stability, trying to avoid chronicity, clinical status perpetuation and sociofunctional impairment, as well as assessing suicidal risk and perform early prevention interventions.

**Methods:**

PAIC is proposed as an early, intensive, focused and psychotherapeutic intervention led by psychiatrist. Consist in an 8-week program of 30 to 60 minute weekly individual sessions. A total of a 205 patients were attended in PAIC during 2021 and 84,6% of the patients completed the program. Mixed-methods, clinical interviews and measurements using validated self-administrated questionnaires were used: CORE-OM pre/post treatment (“Clinical Outcomes in Routine Evaluation-Outcome Measure”), List of Threatening Experiences, *LTE* and Clinical Global Impression (CGI-I). Changes in CORE-OM Scale were analysed using t-student test and a descriptive analysis was used for CGI-results.

**Results:**

CORE-OM (“Clinical Outcomes in Routine Evaluation-Outcome Measure”) showed improvement in all of four dimensions: subjective well-being (TW), problems/symptoms (PT), general function (TF) and risk (TR).

Perception of clinical improving measured by CGI was 81,6%. There were no cases of clinical worsening.

**Conclusions:**

We conclude that intensive and early programs are effective reducing the intensity of symptoms and the level of disability in people who are experiencing a psychological crisis. Also, it supports primary health care as well as helps to avoid saturation of specialized system.

**Disclosure of Interest:**

None Declared

